# MnFe_2_O_4_ Nanoparticles Synthesized via *Citrus paradisi* Extract: A Novel Green Platform for High-Sensitivity Electrochemical Sulfite Detection

**DOI:** 10.3390/foods15183260

**Published:** 2026-09-15

**Authors:** Tomas Tapia-Muñoz, Martina Tapia-Aranda, Ronald Nelson, Arnoldo Vizcarra, Cristofer Gaete-Collao, Mariña Castroagudín, Erico R. Carmona, Aliro Villacorta, Lucas Patricio Hernández-Saravia

**Affiliations:** 1 Laboratorio de Bionanomateriales, Facultad de Recursos Naturales Renovables, Universidad Arturo Prat, Campus Huayquique, Av. Arturo Prat s/n, Iquique 1100000, Chile; ttapiam@estudiantesunap.cl (T.T.-M.); matapiaa@estudiantesunap.cl (M.T.-A.); crgaetec@estudiantesunap.cl (C.G.-C.); avillaco@unap.cl (A.V.); 2 Núcleo de Investigación Aplicada e Innovación en Ciencias Biológicas, Facultad de Recursos Naturales Renovables, Universidad Arturo Prat, Campus Huayquique, Av. Arturo Prat s/n, Iquique 1100000, Chile; 3 Departamento de Química, Facultad de Ciencias, Universidad Católica del Norte, Avda. Angamos 0610, Antofagasta 1270709, Chile; rnelson@ucn.cl (R.N.); marina.castroagudin@ce.ucn.cl (M.C.); 4 Instituto de Alta Investigación, Universidad de Tarapacá, Arica 1100000, Chile; arnoldo.vizcarra.rojas@gmail.com

**Keywords:** eco-friendly, *Citrus paradisi*, sulfite, MnFe_2_O_4_NPs

## Abstract

Developing sustainable paradigms for nanomaterial synthesis is essential to reduce the environmental impact of conventional chemical routes. Herein, we report a green, phytochemically mediated synthesis of MnFe_2_O_4_NPs using aqueous grapefruit (*Citrus paradisi*) peel extracts. The bioflavonoid and polyphenolic constituents functioned as dual-functional biogenic reducing and stabilizing agents. FTIR analysis verified spinel lattice metal–oxygen bonds and surface-bound biogenic groups, while SEM imaging revealed the surface morphology and structural aggregation of the nanoparticles. Once integrated into an electroanalytical platform (MnFe_2_O_4_NPs/GCE), the modified interface exhibited robust electrocatalytic activity toward sulfite oxidation in an acetate buffer (pH = 4.0). Mixed-valence dynamics (Mn^2+^/Mn^3+^ and Fe^2+^/Fe^3+^) significantly amplified anodic currents and lowered the kinetic overpotential. Under optimized chronoamperometric conditions, the sensor demonstrated a wide linear range (1–100 μM, r = 0.999), a low detection limit (LOD = 0.045 μM), a limit of quantification (LOQ = 0.136 μM) and excellent selectivity against co-existing ionic and organic interferents. This study provides a simple, eco-friendly, and technically robust strategy for fabricating high-performance electrochemical platforms for food and environmental monitoring.

## 1. Introduction

The increasing global demand for advanced functional materials has necessitated a paradigm shift toward sustainable manufacturing processes that minimize the environmental footprint of nanotechnology [1,2]. Conventional synthesis routes for transition metal oxides, such as co-precipitation, hydrothermal treatment, and sol–gel methods, often rely on hazardous chemical reductants (e.g., sodium borohydride or hydrazine) and intensive energy inputs [3,4,5,6]. In alignment with the “Twelve principles of green chemistry,” there is an urgent need to replace these synthetic pathways with biogenic alternatives that utilize renewable resources and non-toxic precursors [7]. Among these, the valorization of lignocellulosic biomass and agro-industrial waste represents an important strategy within the circular economy, transforming low-value residues into high-performance engineering materials [8,9,10].

Among the various transition metal oxides, spinel-type ferrites, particularly manganese ferrite (MnFe_2_O_4_), have emerged as key materials in electrochemical science due to their unique structural properties [11]. MnFe_2_O_4_ typically adopts a mixed spinel configuration where divalent Mn^2+^ and trivalent Fe^3+^ ions partition across both tetrahedral and octahedral interstitial sites [12,13]. This specific distribution of cations fosters a rich environment of mixed-valence states, giving rise to remarkable chemical stability, high magnetic permeability, and outstanding intrinsic electrocatalytic properties [13,14,15,16]. These attributes make manganese ferrites exceptionally well-suited for building chemically modified electrodes [6]. However, the electrocatalytic performance of these systems is heavily dependent on surface morphology and the concentration of oxygen vacancies, factors that remain highly challenging to regulate or optimize using standard “top-down” or “bottom-up” chemical protocols [17].

To address these challenges, we present a “benign-by-design” strategy for fabricating MnFe_2_O_4_NPs utilizing aqueous grapefruit (*Citrus paradisi*) peel extracts [18,19,20]. The rich phytochemical profile of *Citrus paradisi* is uniquely suited for eco-friendly because grapefruit peel contains high concentrations of bio-flavonoids (such as naringin and hesperidin), as well as polyphenols and organic acids [21]. These biogenic compounds act as a dual-functional system during nanoparticle synthesis. First, they serve as natural reducing agents that drive the nucleation of the ferrite lattice from metal precursors under mild, eco-friendly conditions [22,23,24]. Second, they function as capping agents that exert steric control over crystal growth [25,26,27]. This natural capping mechanism prevents nanoparticle agglomeration and introduces active organic functional groups onto the nanoparticle surface, which significantly enhances interfacial electron transfer processes during electroanalytical reactions [28].

The analytical utility of these biogenically prepared nanomaterials is demonstrated here through the electrochemical detection of sulfite, an important target analyte in environmental monitoring and food safety [29,30]. Although sulfites are widely used as preservatives, antioxidants, and antimicrobial agents in the food, beverage, and pharmaceutical sectors, their consumption above established regulatory levels (such as those established by the FDA or EFSA) may pose severe health risks, including severe asthmatic reactions and dermatological hypersensitivity [31,32,33]. As a consequence, modern analytical chemistry demands sensing platforms that offer high sensitivity, low limits of detection (LOD) and quantification (LOQ), and more sustainable fabrication approaches [34]. In particular, the viticultural sector requires rapid, straightforward, highly selective, and cost-effective analytical tools for monitoring sulfite concentrations, which is vital for maintaining rigorous quality control across production lines [35]. To address this need, a diverse array of methodologies has been established for quantifying sulfite in complex sample matrices, including enzymatic assays [36], spectrophotometric detection [37], chemiluminescence [38], flow injection analysis [39], capillary electrophoresis [40], volumetric titrations [41], phosphorimetry [42], colorimetric tests [43], spectrofluorimetry [44], and electrochemistry methods [45]. Among these approaches, electrochemical techniques stand out as the most viable candidates for industrial integration, offering superior performance in terms of cost-efficiency, operational simplicity, and adaptability for real-time, on-site monitoring.

Herein, we report the systematic characterization of biogenic MnFe_2_O_4_NPs and evaluate their performance as an electrochemical sensing platform. By integrating green nanotechnology with electroanalytical chemistry, this study demonstrates that *Citrus paradisi*-mediated synthesis yields manganese ferrites with superior electrocatalytic activity toward sulfite oxidation compared to counterparts prepared via traditional chemical routes. This work contributes to the development of more sustainable electrochemical sensing platforms and provides a highly sensitive, sustainable tool for monitoring critical environmental pollutants.

## 2. Materials and Methods

### 2.1. Materials and Reagents

All chemical reagents utilized in this work were of analytical grade and used as received without further purification. Manganese(II) chloride tetrahydrate (MnCl_2_·4H_2_O, ≥98%), iron(III) chloride hexahydrate (FeCl_3_·6H_2_O, ≥97%), sodium hydroxide (NaOH, ≥98%), and sodium sulfite (Na_2_SO_3_, ≥98%) were purchased from Sigma-Aldrich. All aqueous solutions and serial dilutions were prepared using ultrapure water (resistivity 18.2 MΩ·cm) obtained from a Milli-Q water purification system.

### 2.2. Instrumentation and Characterization

Electrochemical measurements were performed using a Biologic SP-150e potentiostat operated via EC-Lab software (V11.72.4). A conventional three-electrode cell configuration was employed, consisting of a bare or modified glassy carbon electrode (GCE, d = 0.07065 cm^2^) as the working electrode, a saturated Ag/AgCl (saturated KCl) reference electrode, and a platinum wire as the counter electrode. The synthesis progress and optoelectronic properties of the biogenic nanoparticles were monitored using UV-Vis spectrophotometry (Merck) over a spectral range of 200–900 nm. Surface functional groups and coordination chemistry of the biomolecule-capped nanomaterials were investigated using Fourier-transform infrared spectroscopy with attenuated total reflectance (ATR-FTIR, Jasco FTIR 4X). Field-Emission Scanning Electron Microscopy (FE-SEM, Zeiss Merlin) operated at an accelerating voltage of 5.0 kV and a working distance (WD) of 6.83 mm, using an energy-dispersive X-ray spectrometer equipped with a Silicon Drift Detector (SDD).

### 2.3. Preparation of Grapefruit Peel Extract

The grapefruit (*Citrus paradisi*) peels were collected as agro-industrial organic waste from local fruit juice processing facilities in the Tarapacá Region, Chile. The biogenic extract was prepared from fresh *Citrus paradisi* using a solid–liquid extraction method optimized to recover bioactive polyphenols and flavonoids. The collected peels were thoroughly washed with deionized water to eliminate environmental debris and subsequently dried in a forced-air convection oven at 60 °C for 120 h until constant mass was achieved. The dehydrated biomass was finely ground and sieved to obtain a uniform powder, ensuring a high surface-area-to-volume ratio for extraction. The extraction was performed by dispersing the pulverized peel in deionized water at a solid-to-solvent ratio of 1:20 g/mL [46]. The mixture was heated at 70 °C under continuous magnetic stirring (500 rpm) for 2 h. Afterward, the suspension was vacuum-filtered to separate the phytochemically enriched supernatant from the insoluble lignocellulosic residues [47]. The resulting biogenic extract was stored at −20 °C in dark vessels to prevent photo-oxidative degradation of the active antioxidant constituents (primarily flavonoids and organic acids), preserving its reducing power for the subsequent synthesis.

### 2.4. Eco-Friendly of MnFe_2_O_4_NPs

The synthesis of spinel-type MnFe_2_O_4_NPs was conducted via a green biogenic pathway adapted from Gaete-Collao et al. [48]. Initially, a precursor solution was prepared by dissolving MnCl_2_·4H_2_O and FeCl_3_·6H_2_O in ultrapure water at a 1:2 stoichiometric molar ratio (Mn^2+^:Fe^3+^). The salts were added sequentially under constant magnetic stirring (900 rpm) at room temperature, allowing a 15 min stabilization period between additions to ensure ionic homogeneity. Subsequently, the *Citrus paradisi* peel extract was added dropwise to the metal precursor solution. In this system, the active polyphenolic fractions act as natural reducing agents and steric capping ligands. Nucleation of the mixed-metal oxide phase was induced by adjusting the pH of the mixture to 10.0 by the dropwise addition of a 3.0 M NaOH solution under vigorous stirring. The resulting dark precipitate was aged at 80 °C for 2 h to promote structural stabilization and complete phase formation. After cooling to room temperature, the solid phase was separated by centrifugation, washed repeatedly with ethanol and ultrapure water to remove unreacted precursors and excess biomass, and dried in a vacuum oven at 80 °C for 24 h to yield the purified MnFe_2_O_4_NPs.

### 2.5. Preparation of Modified Electrodes

Prior to surface modification, the GCE electroactive area was polished to a mirror-like finish using a 0.05 µm alumina (α-Al_2_O_3_) slurry on a synthetic microcloth pad. To eliminate any residual abrasive particles or adsorbed species, the polished electrode was cleaned by brief sonication in ultrapure water for 2 min and dried under a gentle stream of nitrogen. To prepare the sensing interface, a stable dispersion of the biogenic nanoparticles was formulated by dispersing 0.1 mg of MnFe_2_O_4_NPs in 1.0 mL of ultrapure water. This mixture was subjected to high-intensity ultrasonication for 60 min to ensure a homogeneous, stable colloid. Finally, the GCE surface was modified by drop-casting a 10 µL aliquot of the optimized MnFe_2_O_4_NPs ink onto the active area of the electrode. The solvent was allowed to evaporate under a controlled atmosphere at room temperature (25 °C), yielding a stable and uniform electrocatalytic film (MnFe_2_O_4_NPs/GCE).

## 3. Results

### 3.1. MnFe_2_O_4_NPs Characterization by SEM and EDS

**Figure 1:**
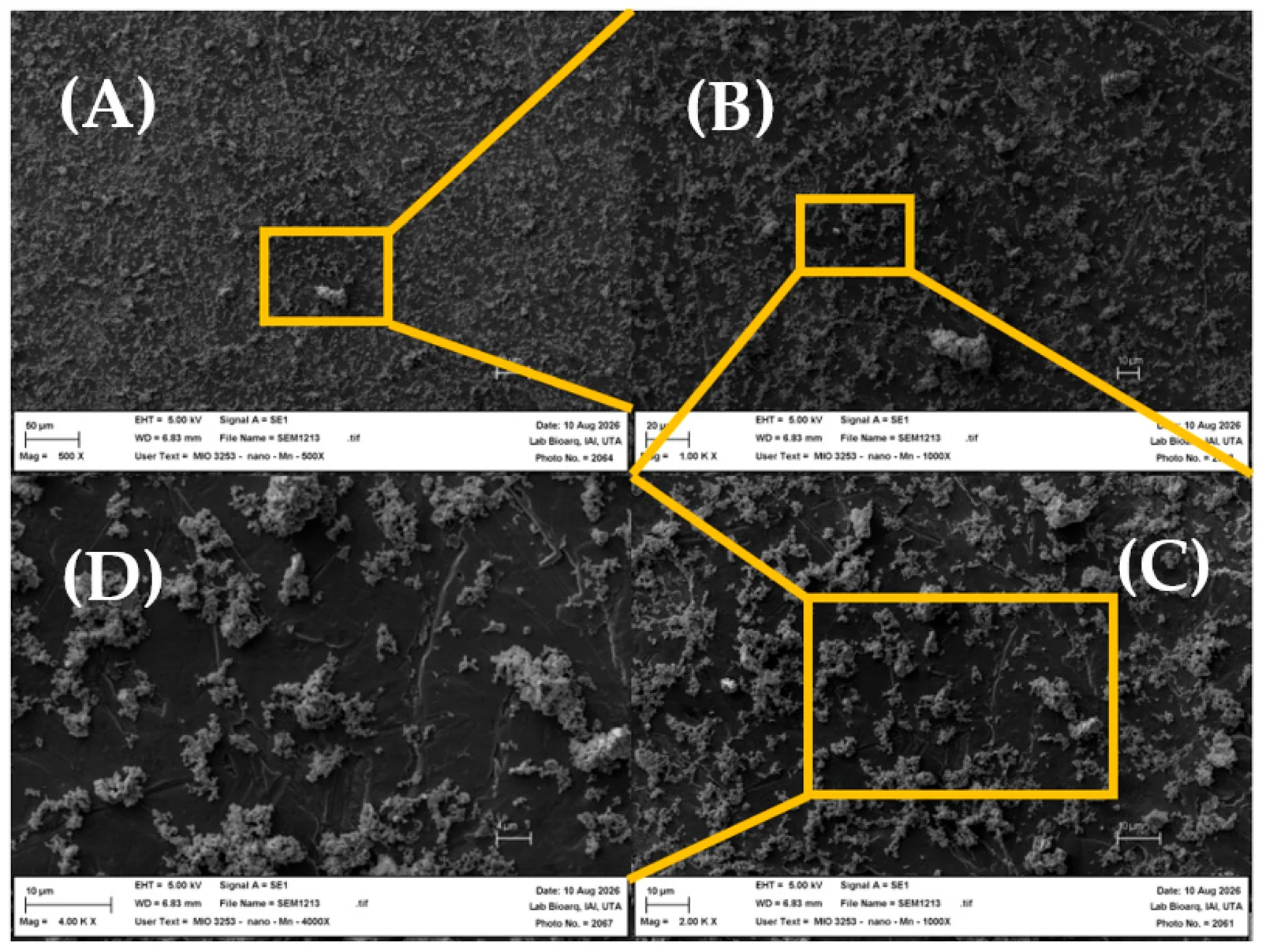
(**A**–**D**) represent FE-SEM microphotographs acquired at progressive magnifications (500×, 1000×, 2000×, and 4000×, corresponding to scale bars of 50 µm, 20 µm, 10 µm, and 4 µm, respectively).

The surface morphology and microstructural features of the biogenically synthesized MnFe_2_O_4_NPs were evaluated using Field-Emission Scanning Electron Microscopy, as presented in Figure 1. The microphotographs reveal a well-defined and relatively uniform distribution of nanoparticles. Although spinel ferrites typically experience magnetic dipole–dipole interactions and Van der Waals attractive forces that drive particle aggregation, the SEM analysis demonstrates that the MnFe_2_O_4_NPs remain stabilized without severe grain sintering. This controlled dispersion is attributed to the steric hindrance provided by the biogenic capping layer derived from the *Citrus paradisi* peel extract.

**Figure 2:**
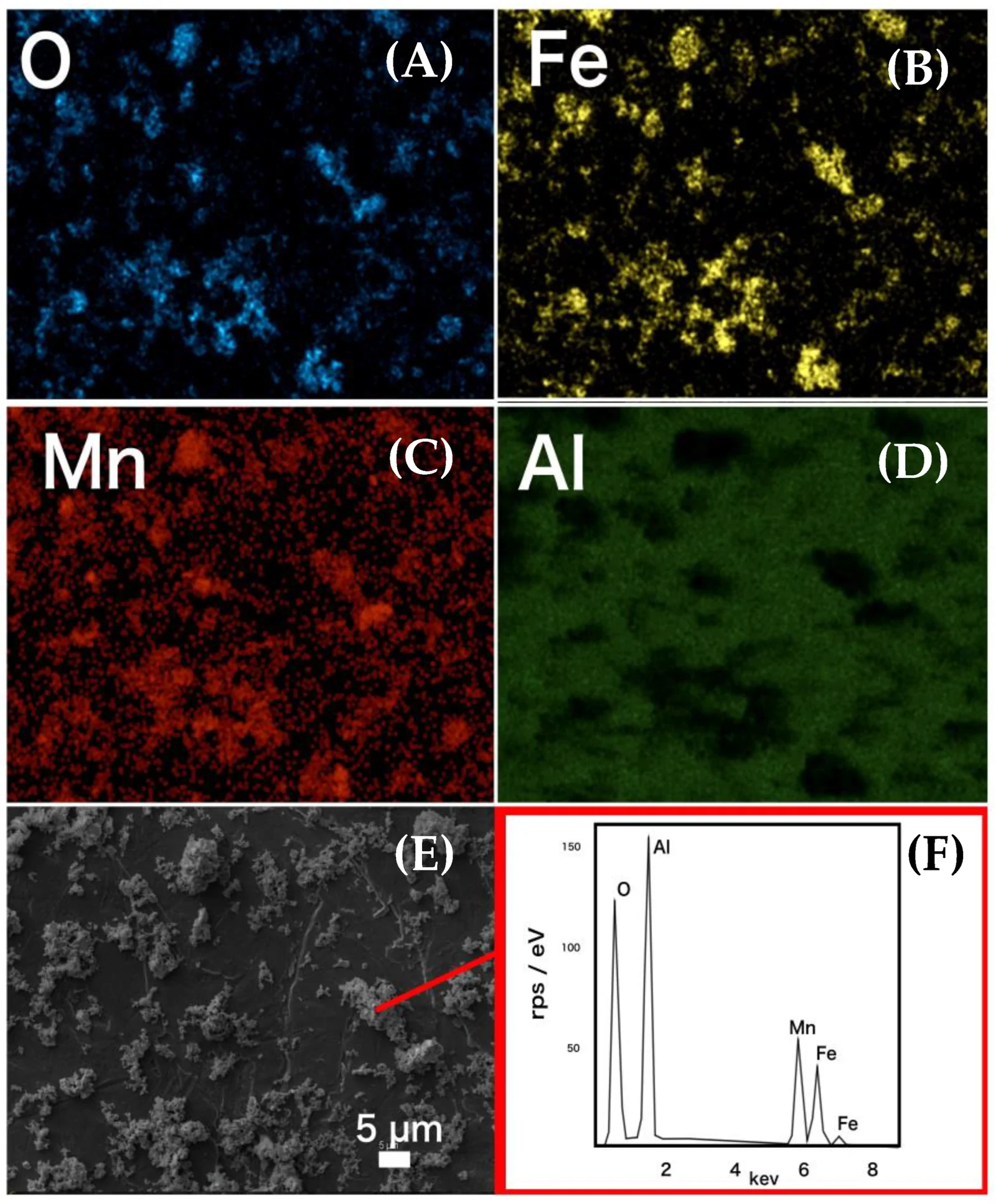
SEM (**A**–**E**) and EDX (**F**) images of MnFe_2_O_4_NPs. The SEM image (**E**) and elemental mapping demonstrate the presence of O (**A**), Fe (**B**), Mn (**C**), and Al (**D**) in the MnFe_2_O_4_NPs.

To complement the morphological observations, the elemental composition and spatial distribution of the constituent elements were investigated by Energy-Dispersive X-ray spectroscopy and elemental mapping, as illustrated in Figure 2. The EDX spectra showed prominent signals corresponding exclusively to manganese (Mn), iron (Fe), and oxygen (O), along with minor signals from aluminum (Al) and sodium (Na), which were attributed to the sample holder and the neutralizing agent used during synthesis, respectively.

The simultaneous detection of Mn, Fe, and O confirms the successful stoichiometric agreement within experimental EDX accuracy of the manganese ferrite phase. In addition, the substantial carbon signal detected on the nanostructure surface corroborates the presence of organic remnants and biopolymer backbones, such as bio-flavonoids and polyphenols, acting as capping ligands from the *Citrus paradisi* extract. Furthermore, the elemental mapping microphotographs (Figure 2) display a highly homogeneous spatial distribution of Mn, Fe, and O across the scanned area. This uniform distribution confirms the chemical homogeneity of the phase and underscores the efficacy of the biogenic reductants in coordinating the mixed-metal oxide lattice at a nanometric scale.

### 3.2. FTIR Spectrum and XRD of the Synthesized MnFe_2_O_4_NPs

**Figure 3:**
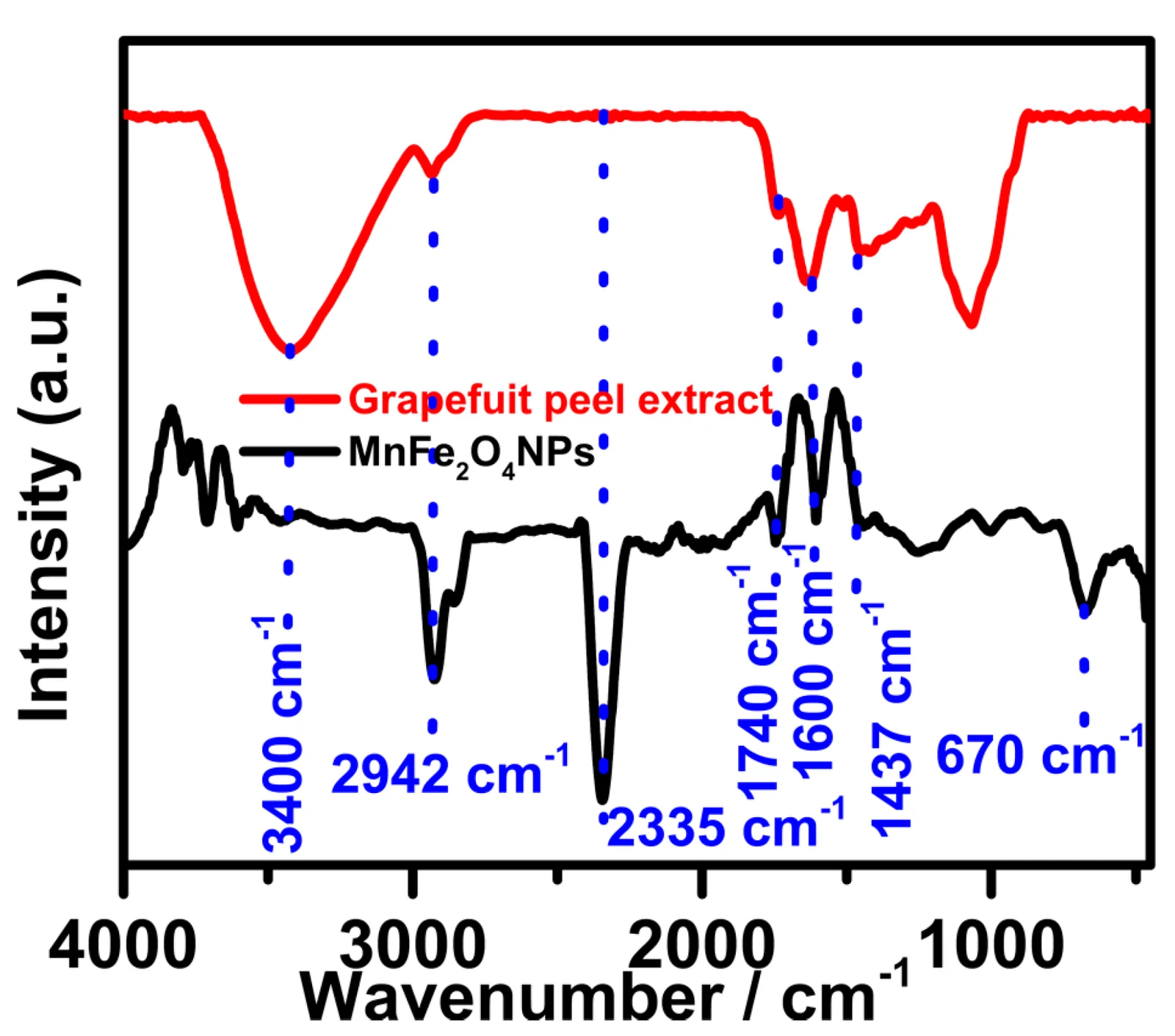
FTIR spectra of MnFe_2_O_4_NPs synthesized using *Citrus paradisi* extract.

The FTIR spectra of the biogenic MnFe_2_O_4_NPs and the aqueous *Citrus paradisi* peel extract are shown in Figure 3, confirming both the successful formation of the spinel metal oxide lattice and the capping role of the phytochemical components. For the synthesized MnFe_2_O_4_NPs, the characteristic vibration of the spinel framework is evidenced by the prominent absorption band centered at approximately 670 cm^−1^, which is assigned to the intrinsic stretching vibrations of the metal–oxygen bonds (Mn–O and Fe–O) [49,50] in the tetrahedral interstitial sites (M–O) [51]. The presence of surface-coordinated hydroxyl groups is indicated by the bending vibrations of metal-bound hydroxyl moieties (Mn–OH and Fe–OH) at approximately 1600 cm^−1^ [51], alongside a broad, intense absorption band spanning 3400 to 3700 cm^−1^ corresponding to the stretching vibrations of physically adsorbed water and hydroxyl (O–H) groups [52].

Additionally, the FTIR profile of the *Citrus paradisi* extract reveals the rich abundance of bioactive reducing and stabilizing agents. The broad band at 3400 cm^−1^ is attributed to the O–H stretching vibrations of polyphenols and bio-flavonoids (e.g., naringin) [52], while the peak at 1740 cm^−1^ is assigned to the stretching vibrations of carbonyl groups (C=O) from organic acids [53]. The twin bands observed at 1437 cm^−1^ and 1600 cm^−1^ correspond to the symmetric and antisymmetric stretching of carboxylate groups (COO^−^), confirming the deprotonated state of the organic acids involved in the metal ion reduction. The presence of aliphatic biomolecules on the nanoparticle surface is further supported by the C–H stretching vibration at 2942 cm^−1^ [54] and the conjugated carbon–carbon (C=C) stretching at approximately 2355 cm^−1^ [46,55,56], which is commonly observed in biogenically prepared metallic oxides and confirms the successful surface capping and steric stabilization of the MnFe_2_O_4_NPs by the *Citrus paradisi* phytomolecules, as can be seen in Table 1.

The structural features, phase integrity, and long-range atomic ordering of the green synthesized MnFe_2_O_4_NPs mediated by *Citrus paradisi* peel extract were comprehensively evaluated via XRD (Figure S1). As illustrated in the diffraction profile, the synthesized nanostructures exhibit well-defined, sharp, and high-intensity Bragg reflections set against a flat baseline, confirming the highly crystalline nature of the material. Distinct diffraction signatures observed at 2 theta positions of 18.1°, 29.7°, 35.0°, 36.6°, 42.5°, 52.7°, 56.2°, 61.7°, and 72.9° correspond strictly to the (1 1 1), (2 2 0), (3 1 1), (2 2 2), (4 0 0), (4 2 2), (5 1 1), (4 4 0), and (5 3 3) crystallographic planes, respectively. These reflection patterns index seamlessly to the face-centered cubic inverse-spinel lattice belonging to the Fd3m space group, showing excellent agreement with standard crystallographic database references (JCPDS card N° 01–074-2403) [57]. Crucially, no characteristic reflections corresponding to secondary impurity phases, such as unreacted single-metal oxides like Fe_3_O_4_ or Mn_3_O_4_, were detected. This phase purity confirms that the phytochemical constituents in the *Citrus paradisi* extract successfully facilitated a complete solid-state phase transformation into a homogeneous, single-phase binary spinel matrix without compromising structural integrity. The unit-cell lattice parameter (α) for the cubic system was determined from the experimental interplanar distances (d_hkl_) using the characteristic relationship $\alpha =d_{hkl}\sqrt{h^{2}+k^{2}+l^{2}}$. Based on the high-intensity (3 1 1) plane (d_(3 1 1)_ = 2.562 Å), the refined lattice constant was calculated to be α = 8.497 Å, which aligns remarkably well with the theoretical value reported for bulk manganese ferrite ($\alpha_{0}$ = 8.51 Å). The subtle lattice contraction observed is typically associated with nanometer-scale surface stress and cation distribution across tetrahedral (A) and octahedral (B) interstitial sites. Furthermore, the average coherent scattering domain size (crystallite size, D) was estimated using the classical Debye–Scherrer relationship:

$$D=\frac{K\times \lambda }{\beta *cos\theta }$$

where K represents the crystallite shape factor (0.90), λ is the incident X-ray wavelength corresponding to CuKα radiation (0.15406 nm), β denotes the full width at half maximum of the predominant reflection in radians, and θ is the Bragg diffraction angle. Based on the line broadening of the primary (3 1 1) reflection centered at 2θ = 35.0° (θ = 17.5°, θ = 0.00512 rad), the mean crystallite size was calculated to be 28.4 nm. This nanometer-scale domain size underscores the vital role of the biogenic polyphenols and flavonoids in controlling crystal growth kinetics, constraining domain propagation, and preventing severe particle agglomeration during the eco-friendly route.

### 3.3. Electrochemical Surface Area of MnFe_2_O_4_NPs/GCE

**Figure 4:**
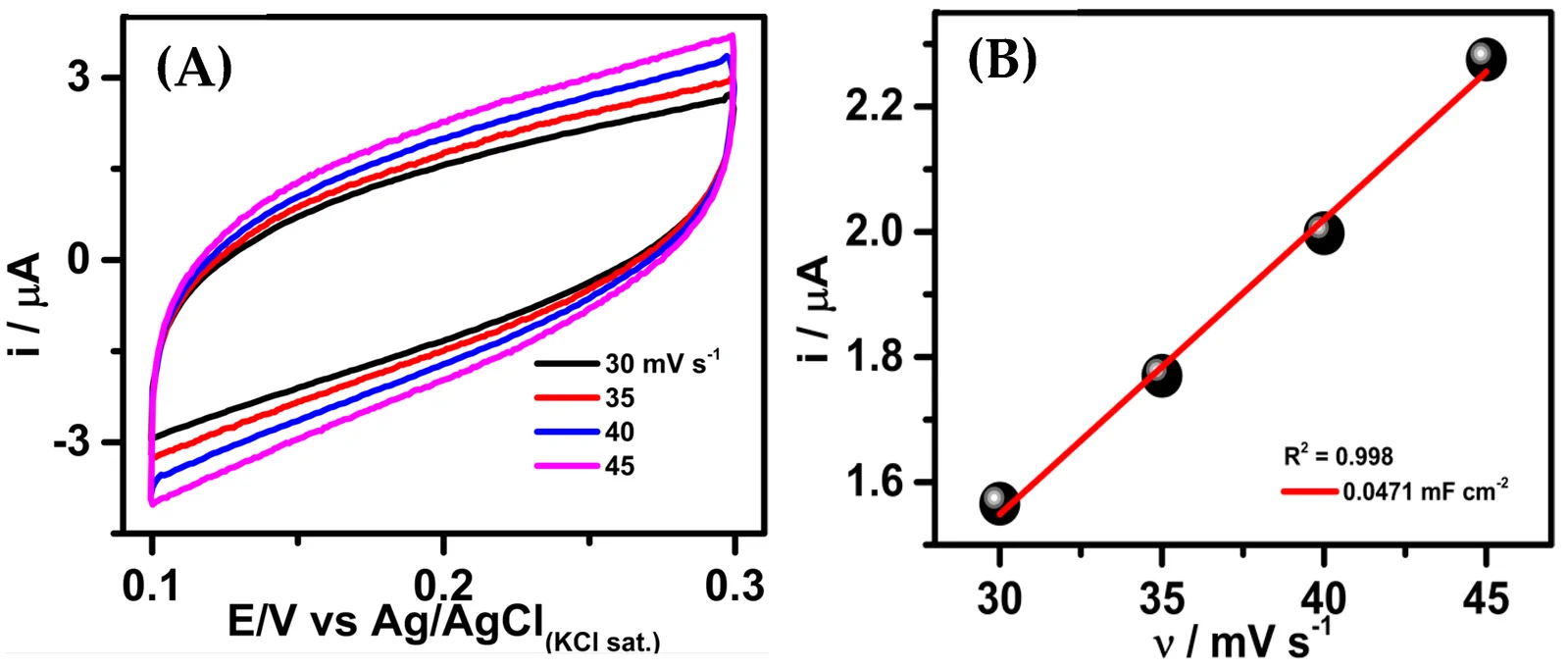
(**A**) ECSA; (**B**) C_dl_ plot for MnFe_2_O_4_NPs.

The outstanding electrocatalytic performance of the developed platform is closely linked to its high density of exposed, highly accessible active sites on the electrode interface. Indeed, a larger electrochemical active surface area (ECSA) directly scales with the abundance of these catalytic sites, substantially boosting overall interfacial charge-transfer efficiency. To quantitatively evaluate this parameter, the double-layer capacitance (C_dl_) was determined by recording cyclic voltammograms (CVs) within the non-Faradaic potential window at progressively increasing scan rates ranging from 30 to 45 mV s^−1^ (Figure 4). The capacitive current densities (j) were measured from the center of this potential region and plotted against the scan rate, yielding a highly linear relationship.

From the slope of this linear fit, the C_dl_ for the MnFe_2_O_4_NPs modified electrode was calculated to be 0.0471 mF cm^−2^. The ECSA was subsequently estimated using the standard relationship:

$$ECSA=\frac{C_{dl}}{C_{s}}$$

where C_s_ represents the specific capacitance of a flat, ideal electrode surface under identical electrolyte conditions, and was assumed to be 0.040 mF cm^−2^ in accordance with established literature standards [58]. Based on this framework, the biogenic MnFe_2_O_4_NPs interface exhibited an outstanding ECSA of 1.1781 cm^2^. This expanded electrochemically active area facilitates rapid electron transfer kinetics and provides a vast number of catalytic coordinates, justifying the enhanced performance of the sensor towards sulfite oxidation [59].

### 3.4. Electrocatalytic Oxidation of Sulfite at MnFe_2_O_4_NPs/CGE

**Figure 5:**
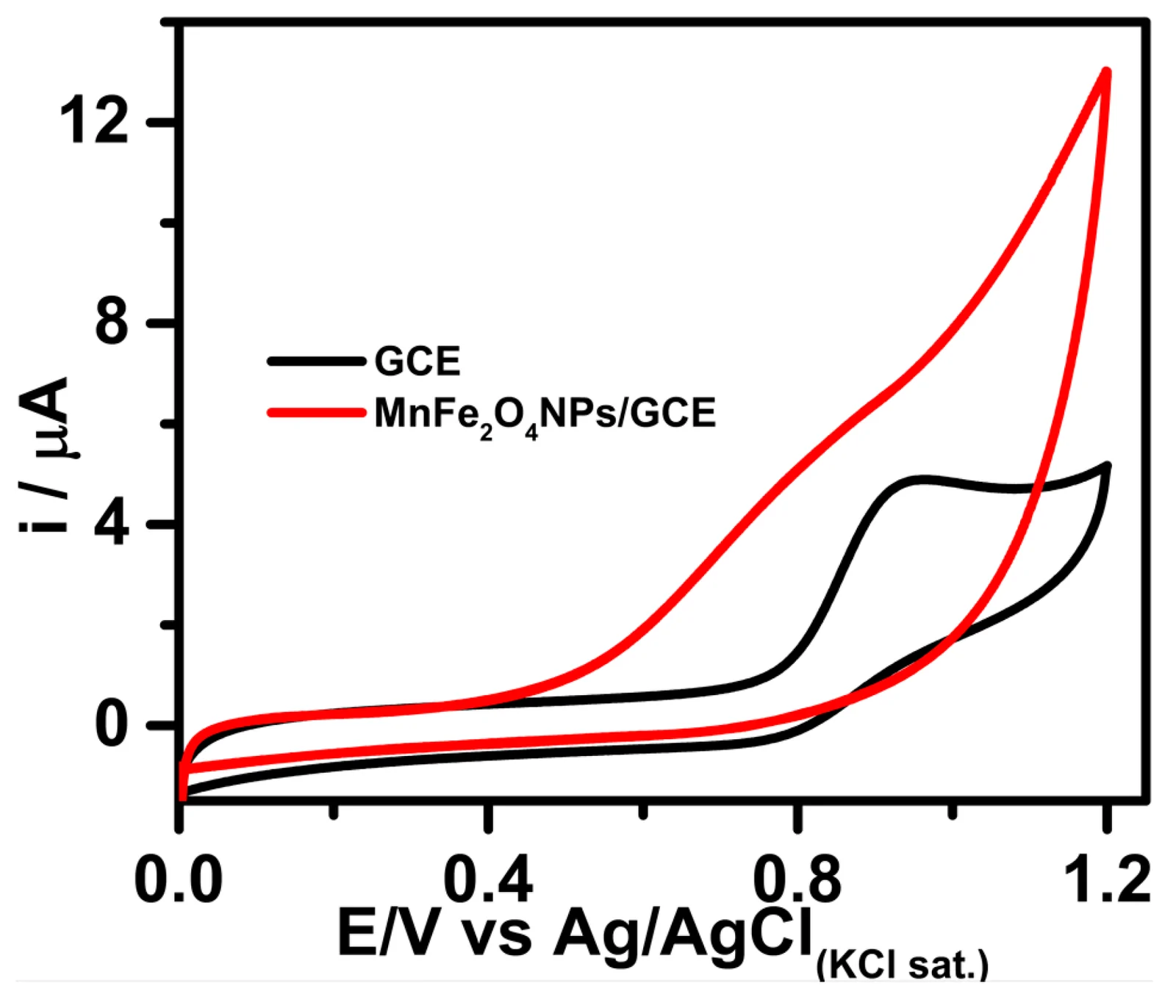
The CVs recorded in a 0.1 M pH = 4 buffer using a GCE (black line) and MnFe_2_O_4_NPs/GCE (red line) in the presence of 1 mM sulfite. Scan rate: 0.1 V s^−1^.

The electrocatalytic performance of the modified MnFe_2_O_4_NPs/GCE toward the oxidation of SO_3_^2−^ was systematically investigated by CV in a 0.1 M acetate buffer supporting electrolyte (pH 4.0). As shown in Figure 5, the unmodified GCE exhibited a negligible electrochemical response both in the absence and presence of 1.0 mM SO_3_^2−^, indicating a low electrochemical activity of the bare carbon interface towards this analyte. In contrast, the MnFe_2_O_4_NPs/GCE interface displayed a sharp, well-defined anodic wave associated with the irreversible electrooxidation of sulfite.

A prominent amplification in the anodic peak current (i_pa_) was achieved upon modifying the electrode surface with the biogenic MnFe_2_O_4_NPs. This enhanced electrocatalytic response is primarily attributed to the synergistic effects arising from the mixed-metal spinel framework. The incorporation of biogenic MnFe_2_O_4_NPs accelerates the interfacial charge-transfer kinetics, which is ascribed to the high current of accessible catalytically active coordinates and the co-existence of multivalent transition metal redox pairs (Mn^2+^/Mn^3+^ and Fe^2+^/Fe^3+^). These mixed-valence states effectively lower the activation energy barrier for the transition of SO_3_^2−^ to SO_4_^2−^ [37]. Furthermore, the improved electrical conductivity and the rich surface chemical properties of the biogenic manganese ferrite nanoparticles provide a thermodynamically favorable pathway for the adsorption and subsequent oxidation of sulfite species, highlighting the superior electrocatalytic advantages of biogenic mixed-metal oxides over conventional single-metal oxide systems.

### 3.5. Effect of pH on the Oxidation Capacity of the MnFe_2_O_4_NPs/GCE

**Figure 6:**
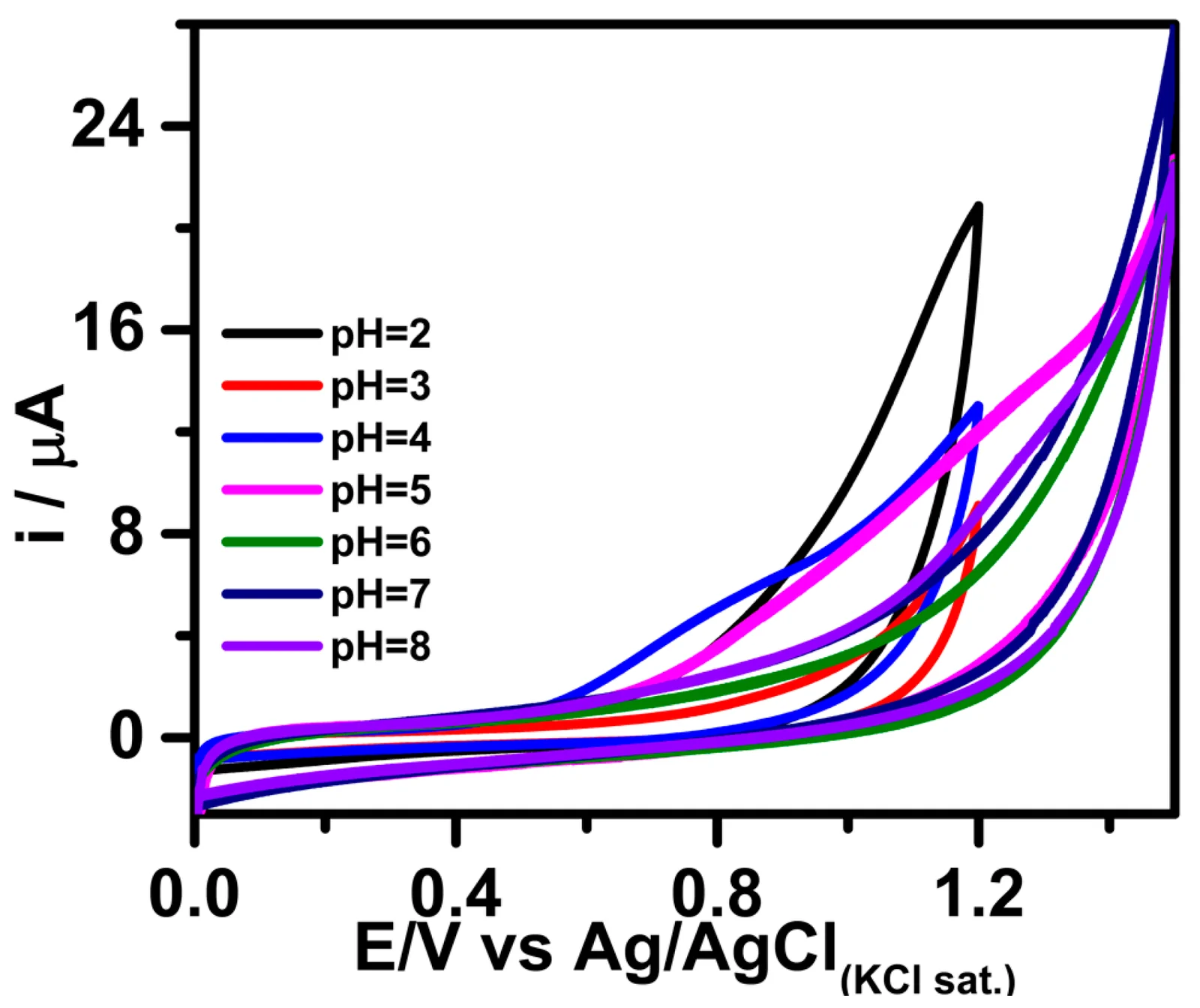
Influence of solution pH on the electrochemical oxidation of sulfite. Cyclic voltammograms recorded at a scan rate of 0.1 V s^−1^.

The effect of the supporting electrolyte pH on the electrocatalytic oxidation of SO_3_^2−^ at the MnFe_2_O_4_NPs/GCE was systematically investigated to optimize analytical sensitivity. CV was conducted over a pH range of 2.0 to 8.0 to evaluate the proton-dependent thermodynamics and kinetics governing the oxidation process (Figure 6).

As the pH increased, the anodic peak potential (E_pa_) shifted toward less positive values, confirming the direct involvement of protons in the rate-determining electrochemical step. The maximum i_pa_ and optimal voltammetric peak symmetry were observed at pH 4.0. At lower pH values (pH < 4.0), the decline in oxidation response is attributed to the increased prevalence of protonated sulfite species (pK_a2_ ≈ 7.2) into less electroactive bisulfite (HSO_3_^−^) forms, along with potential acid-induced instability of the spinel oxide matrix. At higher pH values, the current decreased, likely due to electrostatic repulsion and altered intermediate stability. Consequently, pH 4.0 was chosen as the optimal pH for all subsequent electroanalytical measurements.

The electro-oxidation of SO_3_^2−^ proceeds via a two-electron, proton-coupled charge transfer mechanism. The biogenic MnFe_2_O_4_NPs framework efficiently drives this transformation through the exposed multivalent redox couples (Mn^3+^/Mn^2+^ and Fe^3+^/Fe^2+^) on the spinel surface, which effectively lower the activation overpotential and accelerate intermediate electron transfer.

### 3.6. Calibration Curve for Sulfite Obtained with the MnFe_2_O_4_NPs/GCE Sensor

**Figure 7:**
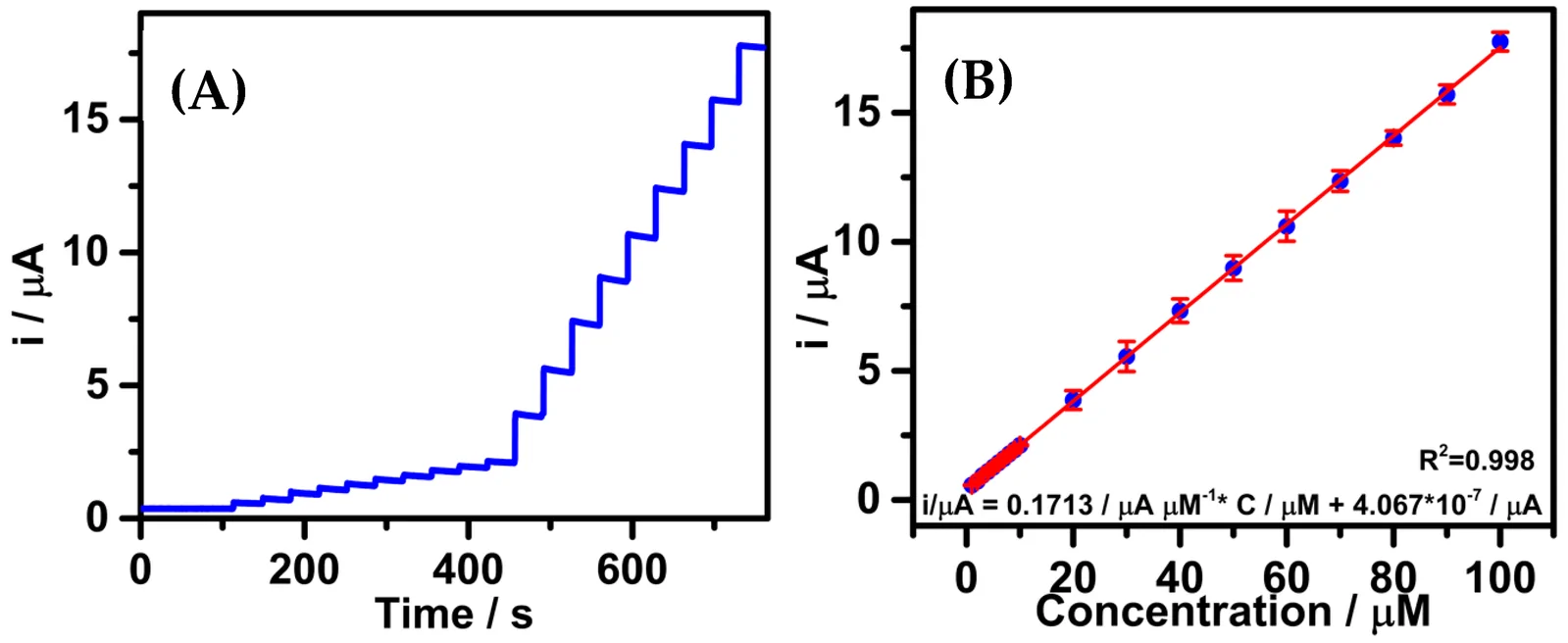
Amperometric responses corresponding to consecutive additions of standard solutions of sulfite 0.1 M pH = 4.0 solution (buffer) using the MnFe_2_O_4_NPs/GCE modified electrode (**A**). Calibration plot in the range 1 μM to 100 μM sulfite (**B**).

The calibration curve was constructed via hydrodynamic amperometry in a stirred 0.100 M acetate buffer solution (pH 4.0) at an applied potential of 0.85 V vs. Ag/AgCl. Successive aliquots of a freshly prepared standard sulfite solution (SO_3_^2−^) were injected under continuous magnetic stirring (300 rpm). The chronoamperometric response of the modified electrode demonstrated dynamic linear behavior over a wide concentration dynamic range of 1–100 μM (Figure 7A), exhibiting high linearity with a determination r of 0.999 (Figure 7B). The LOD and LOQ were statistically derived using the conventions LOD = 3.3σ/m and LOQ = 10σ/m [60,61], where σ represents the standard deviation of ten consecutive blank signal measurements and m corresponds to the slope of the linear regression curve. The calculated LOD and LOQ values were 0.045 μM and 0.136 μM, respectively. Sensor repeatability and reproducibility was evaluated across ten independently prepared MnFe_2_O_4_NPs/GCE, resulting in a relative standard deviations (RSD) of 1.58% and 2.14%, indicating robust electrode fabrication fidelity. The electroanalytical sensitivity, defined as the response current per unit concentration within the linear range, was determined to be 0.1713 µA µM^−1^. To compare the analytical performance of the MnFe_2_O_4_NPs/GCE electrocatalytic platform, its dynamic range, detection limit, and sensitivity were compared against previously reported electrochemical sulfite sensors (Table 2). The developed sensor demonstrated superior electroanalytical performance, exhibiting a lower LOD than most existing platforms in the literature.

### 3.7. Reproducibility, Selectivity, and Stability Studies MnFe_2_O_4_NPs/GCE Sensor

**Figure 8:**
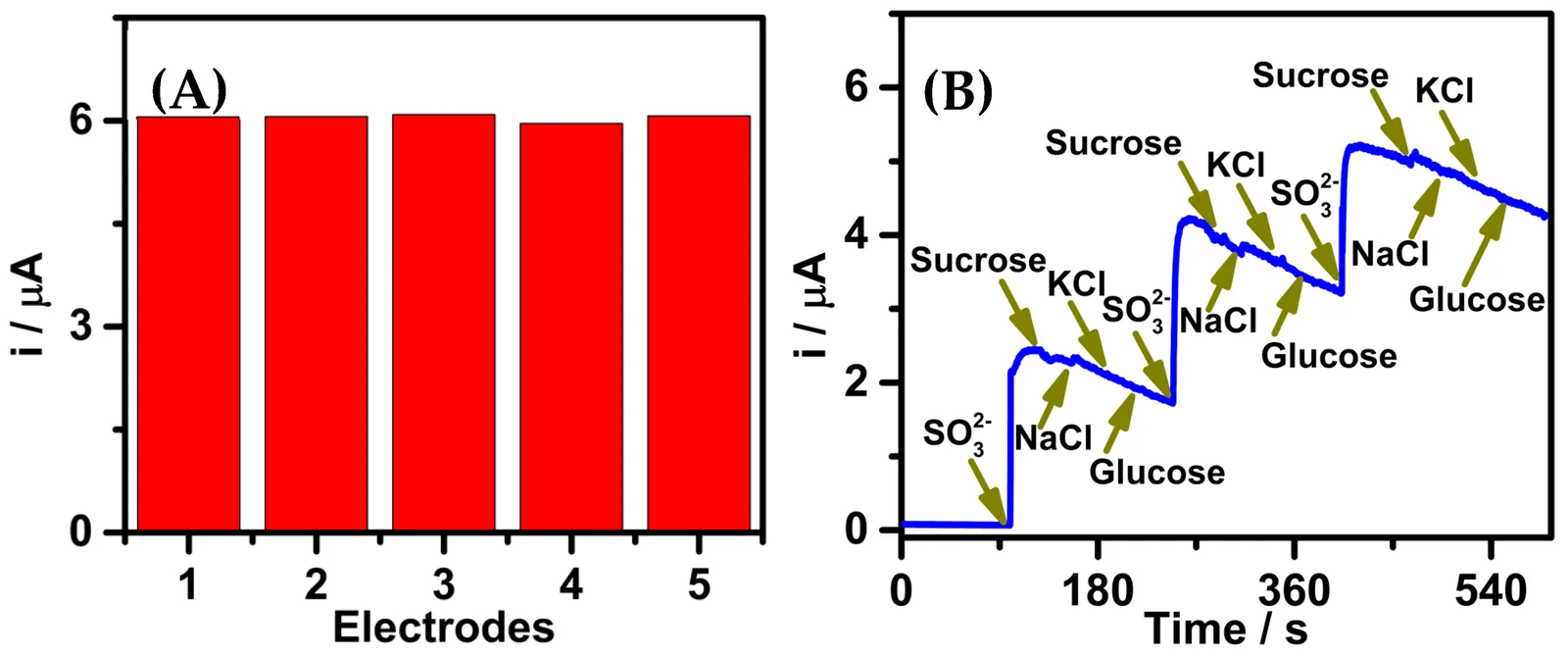
Cyclic voltametric responses for 5 different MnFe_2_O_4_NPs/GCE sensors to 1 mM sulfite (**A**). Effect of the successive addition of 1 mM of Sucrose, NaCl, KCl, and Glucose on the amperometric response used for the reduction of 10 µM sulfite in pH = 4 buffer (**B**).

To evaluate the analytical reliability and practical applicability of the biogenic MnFe_2_O_4_NPs/GCE platform for sulfite determination, key performance parameters were systematically evaluated following Association of Official Agricultural Chemists (AOAC) validation criteria [44]. The sensor was subjected to rigorous electroanalytical testing to assess sensor-to-sensor reproducibility and anti-interference selectivity within complex sample matrices. The sensor-to-sensor fabrication reproducibility was evaluated by recording CV responses across five independently prepared MnFe_2_O_4_NPs/GCE modified electrodes in the presence of 1.0 mM sulfite (pH = 4.0 buffer). As depicted in Figure 8A, the anodic peak current responses displayed remarkable consistency, yielding a low RSD of 2.14%. This low variance confirms high batch-to-batch operational fidelity and verifies the robustness of the drop-casting fabrication protocol. Selectivity constitutes a decisive criterion for real-sample application. The anti-interference capability of the MnFe_2_O_4_NPs/GCE interface was investigated by chronoamperometry at an applied potential of +0.85 V in 0.1 M acetate buffer. Following the steady-state current response obtained after the addition of by 10 μM sulfite, potential interferents, specifically sucrose, NaCl, KCl, and glucose, were sequentially added at a 100-fold molar excess (1.0 mM) (Figure 8B) [74,75,76]. The consecutive additions of these non-target organic and ionic species caused no discernable current fluctuations or signal attenuation. These findings unequivocally confirm the superior catalytic selectivity and structural resilience of the proposed MnFe_2_O_4_NPs/GCE sensor toward sulfite oxidation under the optimized experimental conditions (0.85 V).

### 3.8. Application of the Methods in the Analysis of Foods Samples

To evaluate the practical applicability and analytical accuracy of the engineered MnFe_2_O_4_/GCE platform in real matrices, the electroanalytical sensor was deployed for the direct quantification of sulfite across various commercial liquid and beverage specimens, including tea, mate drink and lemon juice commercial beverages. Amperometric measurements were systematically executed under steady-state hydrodynamic conditions at an optimized polarization potential of 0.85 V vs. Ag/AgCl. Prior to standard addition, non-fortified real specimens exhibited no baseline sulfite signal, confirming that endogenous concentrations were below the limit of detection. The samples were subsequently spiked with known standard concentrations of sulfite (10, 20, and 30 µM) to evaluate recovery rates and potential matrix interference from complex organic constituents such as polyphenols, pigments, and dissolved solids. As summarized in Table 3, the MnFe_2_O_4_/GCE sensing interface displayed excellent recovery values ranging from 99.2% to 104.1% with RSD strictly below 3.9% across all fortified levels. The rapid, well-defined step-like current responses (i-t) recorded at 0.85 V upon successive analyte additions underscore the high electrocatalytic activity, rapid charge-transfer kinetics, and excellent anti-fouling stability of the nanostructured manganese ferrite matrix in complex liquid environments. Overall, these results confirm that the MnFe_2_O_4_/GCE modified electrode represents a reliable, robust, and highly sensitive analytical tool for routine food quality control and regulatory enforcement of sulfite additives.

## 4. Conclusions

In this study, a novel, eco-friendly, and sustainable biogenic route was successfully established for the synthesis of spinel-type MnFe_2_O_4_NPs utilizing aqueous grapefruit peel extract. The bioactive polyphenolic and flavonoid constituents within the agro-industrial waste extract functioned effectively as dual-purpose reducing and steric capping agents, facilitating the formation of phase-pure, well-dispersed nanostructures without requiring toxic chemical reagents or hazardous organic solvents. When integrated onto a glassy carbon electrode surface (MnFe_2_O_4_NPs/GCE), the biogenic nanocomposite demonstrated exceptional electrocatalytic activity toward the irreversible oxidation of sulfite in aqueous media (pH = 4.0). The mixed-valence dynamics (Mn^2+^/Mn^3+^ and Fe^2+^/Fe^3^) inherent to the spinel lattice significantly accelerated interfacial charge-transfer kinetics, providing a high ECSA (1.1781 cm^2^) and effectively lowering the kinetic overpotential for sulfite oxidation. Under optimized chronoamperometric conditions, the sensing platform displayed outstanding electroanalytical features, including a wide linear range (1–100 μM), a remarkably low LOD (0.045 µM), high sensitivity (0.1713 µA μM^−1^), superior operational repeatability (RSD = 1.58%), and robust anti-interference selectivity in the presence of excess ionic and organic matrix constituents. Overall, this work demonstrates the successful valorization of citrus processing by-products into high-value functional nanomaterials, providing a simple, cost-effective, and green electroanalytical tool well-suited for routine food safety inspection, quality control, and regulatory compliance monitoring of sulfite additives in agricultural and food products.

## Figures and Tables

**Table 1:** FTIR analysis of MnFe_2_O_4_NPs and *Citrus paradisi* extract containing possible functional groups.

MnFe_2_O_4_ NPs Wavenumber (cm^−1^)	Functional Groups	*Citrus paradisi* Extract Wavenumber (cm^−1^)	Functional Groups
3400	O–H	3400	O–H
2942	C–H	2942	C–H
2335	C=C		
1740	C=O	1740	C=O
1600	COO^−^	1600	COO^−^
1437	COO^−^	1437	COO^−^
670	Mn–O Fe–O		

**Table 2:** Electrochemical performance of different modified electrodes for sulfite sensing.

Electrode	Linear Range (μM)	LOD (μM)	LOQ (μM)	Sensitivity (µA µM^−1^)	Sample	Ref.
AgNPs/PPy@ PEDOT:PSS/SLT	1–130 ng mL^−1^	0.29 ng g^−1^	—	1.510 μA ng^−1^ mL	Milk and rice flour	[62]
CeO_2_ NPs/SPCE	0.08–870.0	0.023	0.077	0.043	River water and Tap water	[63]
*p*-ADPA-4-ATP-Au/GC	5.0–160	1.5	5	0.0176	Biscuit and soup	[64]
PPY-CHI/GCE	50–1100	0.21	0.7	15.28 μA μM^−1^ cm^−2^	Orange Juice, Malt drink and Human serum	[65]
AgNPs@PANI/rGO/GCE	2.7–24.4	77 nM	25.6	128 μA μM^−1^ cm^−2^	Drinking water, Tap water and River water	[66]
La^3+^-doped Co_3_O_4_ nanocubes/SPE	0.7–1000.0	0.09	0.29	0.0481	Tap water, River water and Waste water	[67]
polyCoFeRu/GCE	6.6–178.17	0.06	0.20	6.98 μA mM^−1^	Tap water, Rain Water and River Water	[68]
Cs-IL/MWCNT@GCE	70–20,200	1.83	6.08	10.5 μA mM^−1^	Raisins and apricots	[69]
Cu(L^NO2^)_2_/GCE	10.00–5000	0.75	2.27	7.43	White wine and red wine	[45]
NiCo_2_O_4_/rGO/GCE	5–4850	3 nM	12 nM	0.32341 μA μM^−1^ cm^−2^	Water, grapes, apple juice	[70]
CoHCF-BLPE	4–128	1.74	5.8	0.008	Tomato Ketchup, Wine, Jam, and Dry grapes	[71]
CPE/Au-Si4Pic^+^Cl^−^	2.54–48.6 mg L^−1^	0.88 mg L^−1^	2.68 mg L^−1^	0.353 μA mg^−1^ L	Coconut water and wine	[72]
PGE/BMNPs/CHIT/GA/PPO^IX^	5–80	0.151	0.452	2.18	Juice sample	[73]
MnFe_2_O_4_NPs	1–100	0.045	0.136	0.1713	Tea, lemon juice and mate drink	This Work

**Table 3:** Determination of SO_3_^2−^ in spiked samples using the proposed MnFe_2_O_4_/GCE sensor.

Samples	Added (µM)	Found (µM)	Recovery (%)	RSD (n = 3) (%)
Tea	40	40.3 ± 1.1	101.1	4.7
	50	52.0 ± 1.0	104.1	3.3
	60	60.9 ± 1.7	101.4	4.8
	70	71.0 ± 1.1	101.4	2.7
Mate drink	40	40.6 ± 0.7	101.6	3.1
	50	50.6 ± 0.3	101.2	1.2
	60	61.7 ± 1.9	102.3	5.4
	70	70.6 ± 0.4	100.9	1.1
Lemon juice	40	39.7 ± 0.3	99.2	1.2
	50	50.4 ± 0.2	100.9	0.8
	60	60.4 ± 0.5	100.6	1.5
	70	70.2 ± 0.1	100.3	0.1

## Data Availability

The data presented in this study are available from the corresponding authors upon request, as they must be included in project reports prior to public disclosure.

## Supplementary Materials

The following supporting information can be downloaded at https://www.mdpi.com/article/10.3390/foods15183260/s1, Figure S1: Standard XRD pattern of MnFe_2_O_4_ (JCPDS No.: 01-074-2403) and XRD analysis of MnFe_2_O_4_ NPs.
