# Supplementary material for: MnFe2O4 Nanoparticles Synthesized via Citrus paradisi Extract: A Novel Green Platform for High-Sensitivity Electrochemical Sulfite Detection
Source: Foods. 2026 Sep 15;15(18):3260. doi: 10.3390/foods15183260 (PMC13606314; doi:10.3390/foods15183260)
Supplement: Supplementary file 1 [file foods-15-03260-s001.zip › foods-4547542-supplementary.pdf]

$MnFe_2O_4$ nanoparticles synthesized via *citrus paradisi* extract: A novel green platform for high-sensitivity electrochemical sul-fite detection

Tomas Tapia-Muñoz [1,2], Martina Tapia-Aranda [1,2], Ronald Nelson [3], Arnoldo Vizcarra [4], Cristofer Gaete-Collao [1,2], Mariña Castroagudín [3], Erico Carmona [1*], Aliro Villacorta [1,2] and Lucas Patricio Hernández-Saravia [2*]

[1] Laboratorio de Bionanomateriales, Facultad de Recursos Naturales Renovables, Universidad Arturo Prat, Campus Huayquique, Iquique 1100000, Chile

[2] Núcleo de Investigación Aplicada e Innovación en Ciencias Biológicas, Facultad de Recursos Naturales Re-novables, Universidad Arturo Prat, Av. Arturo Prat s/n, Campus Huayquique, Iquique 1100000, Chile

[3] Departamento de Química, Facultad de Ciencias, Universidad Católica del Norte, Avda. Angamos 0610, An-tofagasta 1270709, Chile

[4] Instituto de Alta Investigación, Universidad de Tarapacá, Arica, Chile

*Correspondence: ecarmona@unap.cl (E.R.C.); luhernande@unap.cl (L.P.H.-S.)

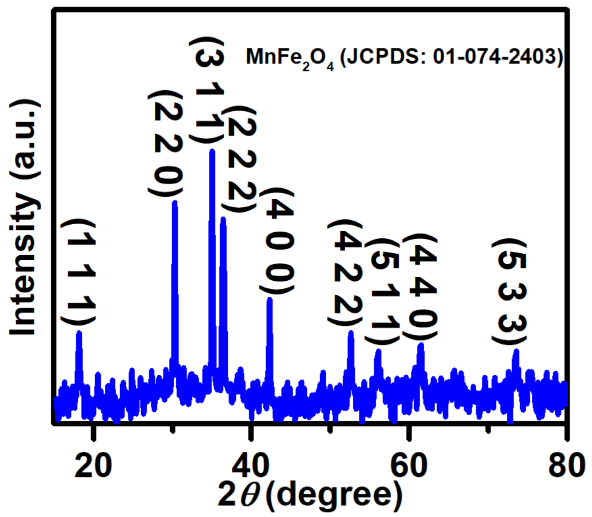

Figure S1. Standard XRD pattern of $MnFe_2O_4$ (JCPDS No.: 01-074-2403) and XRD analysis of $MnFe_2O_4$ NPs.
